# Reasons clinical education directors intend to leave their jobs

**DOI:** 10.1186/s12909-023-04099-2

**Published:** 2023-02-27

**Authors:** Alicia Klein, Katrina Schrode, Lucy Kibe, Gerald Kayingo

**Affiliations:** 1 Graduate School, Bethel University, Saint Paul, MN USA; 2 Department of Psychiatry, Charles R. Drew University of Medicine and Science, Los Angeles, CA USA; 3 College of Science and Health, Charles R. Drew University of Medicine and Science, Los Angeles, CA USA; 4 Physician Assistant Leadership and Learning Academy, Graduate School, University of Maryland Baltimore, Baltimore, MD USA

**Keywords:** Clinical director, Clinical coordinator, Education, Intent to leave, Satisfaction, Burnout

## Abstract

**Objectives:**

The goals of this study were to characterize clinical coordinators compared to other Physician Assistant (PA) faculty, and investigate factors associated with intent to leave their position, institution, and academia in the U.S.

**Methods:**

This was a secondary analysis of data obtained from the 2019 Physician Assistant Education Association (PAEA) Faculty and Directors Survey. We examined bivariate associations with faculty role and conducted multiple logistic regression to identify predictors of intent to leave among clinical directors.

**Results:**

Clinical directors indicated an intention to leave their position more often than other faculty. Factors influencing intent to leave were a lack of satisfaction with professional development and moderate to high levels of burnout. Clinical directors with severe burnout had 27x the odds of intending to leave academia.

**Conclusions:**

Our results suggest why clinical directors intend to leave and underscore the need for professional development opportunities and faculty mentoring. Faculty-centered work arrangements are needed to reduce burnout among clinical directors.

## Introduction

Physician Assistant (PA) programs have had exponential growth globally in recent years; however, recruitment and retention of qualified faculty is emerging as a major limiting step in PA education [1]. PA program faculty are typically PAs who have taken on educational and/or administrative responsibilities, often in addition to maintaining clinical duties. According to the 2019 Physician Assistant Education Association (PAEA) Program Survey, there were 115 unfilled fulltime equivalent (FTE) positions with about 87 (21%) of PA programs reporting vacant FTEs [2]. Clinical Directors^1^ (CD) are key members of the PA faculty in supporting the clinical phase of PA programs. There have been anecdotal reports of high turnover of this group of faculty. This is especially important as issues of clinical sites, preceptors, and clinical coordination have become a daunting task as the number of PA programs continues to rise [2–5]. On a global scale, the shortage of clinical sites has created significant difficulties in clinical coordination such as securing partnerships with health systems [6]. Increased demands in clinical coordination may lead to burnout and dissatisfaction, which ultimately impact faculty morale, retention, and student outcomes. Whether these factors also contribute to intention to leave remains to be established [7, 8]. Literature is still lacking on the factors influencing the CD’s role dynamics.

The goals of this study were to 1) describe features that characterize CDs compared to other faculty, and 2) investigate predictors of intent to leave in CDs compared with other faculty. Findings from this study have far-reaching implications for PA program leadership and faculty teams as well as ensuring desired program outcomes and a qualified PA health workforce. To our knowledge this is one of the first studies that focuses on the CD group in PA programs, their characteristics, and their intent to leave their position, institution, and/or academia.

For the study of faculty turnover and intend to leave their jobs, several theoretical frames were explored for understanding why clinical coordinators are leaving. It can be assumed that an employee’s decision to leave may be influenced by that employee’s perceptions about the desirability and ease of movement. Internal factors such as job satisfaction and organizational commitment influence the desirability of movement whereas external factors such as job market conditions and labor market mobility influence the ease of movement [9]. Another perspective utilizes the theory of vocational choice where satisfaction, stability, and achievement depend on the congruence between one’s personality and environment in which one works [10].

## Methods

This was a secondary data analysis based on raw data obtained from the 2019 PAEA Faculty and Directors Survey. Only PA faculty who have been trained and certified as PAs were included in this study. The survey response rate was 60.5% with representation from 97.9% of all programs. The Institutional Review Board at Charles R. Drew University of Medicine and Science approved the study.

In the current study, we assess the characteristics of CDs and their intention to leave academia, their institution, or their position compared to “other” PA faculty. “Other” PA faculty were defined as all other faculty not holding a CD role, except program directors or above (e.g. department chair, dean, etc) and medical directors who were excluded due to the exceptional conditions of those positions. Adjuncts and those with FTE < 50% were also excluded. We used descriptive, bivariate chi-squared tests and t-tests to compare CDs and other faculty in terms of characteristics and potential predictors of intent to leave their jobs.

Features characterizing CDs that were examined were age, gender, underrepresentation in medicine (URIM) status, years in current position, academic rank, type of school, levels of satisfaction, level of burnout, and intent to leave. Based on the Association of American Medical Colleges definition of underrepresentation in medicine, PAEA categorized those who identify as white or Asian as not under-represented, and all other racial/ethnic groups as under-represented. We excluded type of schools categorized as public/private hybrid or military, due to small numbers.

According to the PAEA survey description, individuals were asked to rate their level of satisfaction with a wide range of factors on a 4-point scale (Not satisfied-Very satisfied). Burnout was assessed using a validated item from the Maslach Burnout Inventory. Burnout is defined in the ICD-11 as a syndrome associated with energy depletion, mental distance, and decreased professional efficiency resulting from chronic workplace stress. The question asked how frequently participants felt burned out from their work on a 7-point scale with response options ranging from Never to Every day.

Intent to leave was characterized by three questions, which captured different levels of the intention. Participants were asked, “In the past 2 years, did you consider leaving your current *position* for another one within the same PA program?”, “In the past 2 years, did you consider leaving your current *institution* for another institution?”, and “In the past 2 years, did you consider leaving *academia* for a different job?”, and responses options were yes or no. We treated each response as a separate outcome.

We performed multiple logistic regression separately among CDs and other faculty to determine predictors of intention to leave at each level (i.e. academia, institution, position), with listwise deletion and correcting for the characteristics mentioned above. Age, burnout, and years in current position were included as continuous variables, while gender, URIM status, academic rank, and type of school were included as categorical variables. Because there were many elements of satisfaction evaluated, to simplify analysis, we categorized satisfaction into four groups: Professional development, Job duties, Wellbeing & benefits, and Support & environment. While we recognize that these are arbitrarily chosen categories, we felt they reflected the broad domains of the factors that were asked about. These factors are summarized in Table 1. For each category of satisfaction, we calculated the average score of the corresponding component factors. The 4 new satisfaction variables were included in the regression models as binary categorical variables, based on the original coding (Marginally satisfied/Not satisfied vs. Satisfied/Very satisfied). We present adjusted odds ratios (AOR), 95% confidence intervals (95% CI), and *p*-values for the regression models. *P* < 0.05 is considered statistically significant for all analyses. All analyses were performed using SAS 9.4.

**Table 1:** Categorization of components of satisfaction

Category	Components
Professional development	Current academic rank
	Faculty development opportunities outside institution (e.g., conferences)
	Faculty development opportunities within institution (e.g., grant workshops)
	Promotion potential
	Research opportunities
	Tenure requirements
Job duties	Job responsibilities
	Teaching workload
	Clinical work arrangement
	Curriculum
Wellbeing & benefits	Autonomy and independence
	Departmental support for work/life balance
	Retirement benefits
	Salary amount
	Schedule flexibility
	Healthcare plan
	Fairness of salary relative to other faculty
Support & environment	Staff support
	Program management/leadership
	Institutional leadership
	Quality of students
	Didactic or clinical teaching environment
	Student to faculty ratio

## Results

### Participant characteristics

Table 2 shows the characteristics of all participants. About 22% of faculty were clinical directors. The mean age of all faculty was 44 years, and on average, participants had been in PA education for 2.6 years, at their program for 2.3 years, and in their position for 1.8 years. The sample was mostly female (73.6%), white (85.8%), and non-URIM (87.8%). Almost 18% of CDs held the rank of instructor/lecturer/other and 16% held the rank of associate professor or professor, while only 11% of other faculty had the rank of instructor/lecturer/other and 22% held the rank of associate or full professor. Although the majority of all participants did not have a doctorate degree (86.6%), CDs held almost half as many doctorates as other faculty.

**Table 2:** Characteristics of CDs compared with other faculty

Characteristic	Total	Clinical Director (CD)	Other Faculty	
	***n*** = 723	***n*** = 156 (21.6%)	***n*** = 567 (78.4%)	
	Mean ± SD	Mean ± SD	Mean ± SD	***p*** value
**Age**	44.2 ± 10.9	44.0 ± 10.3	44.2 ± 9.9	0.801
**Years in PA education**	2.6 ± 1.3	2.6 ± 1.3	2.7 ± 1.3	0.606
**Years at program**	2.3 ± 1.3	2.3 ± 1.3	2.3 ± 1.2	0.489
**Years at current position**	1.8 ± 1.3	1.8 ± 1.3	1.7 ± 1.2	0.557
	**# (%)**	**# (%)**	**# (%)**	
**Type of School**				0.157
Private, non-profit	438 (63.9)	100 (66.7)	338 (63.1)	
Private, for-profit	31 (4.5)	10 (6.7)	21 (3.9)	
Public	217 (31.6)	40 (26.7)	177 (33.0)	
**Gender**				0.389
Male	187 (26.4)	36 (23.7)	151 (27.2)	
Female	521 (73.6)	116 (76.3)	405 (72.8)	
**Race/Ethnicity**				0.968
White	620 (85.8)	131 (84.0)	489 (86.2)	
Asian	15 (2.1)	4 (2.6)	11 (1.9)	
African American	29 (4.0)	6 (3.8)	23 (4.1)	
Hispanic	27 (3.7)	7 (4.5)	20 (3.5)	
NHPI/AIAN	5 (0.7)	1 (0.6)	4 (0.7)	
Other or no answer	27 (3.7)	7 (4.5)	20 (3.5)	
**Under-represented status in medicine**				0.755
Non-UR in medicine	635 (87.8)	134 (90.5)	497 (91.4)	
UR in medicine	88 (12.2)	14 (9.5)	47 (8.6)	
**Tenure status**				0.244
Not tenure track	588 (81.3)	130 (83.3)	458 (80.8)	
Tenure track, not tenured	110 (15.2)	24 (15.4)	86 (15.2)	
Tenured	25 (3.5)	2 (1.3)	23 (4.1)	
**Degree awarded at PA school graduation**				0.017
Certificate or Associate	77 (10.7)	25 (16.0)	52 (9.2)	
Bachelor	182 (25.2)	30 (19.2)	152 (26.9)	
Master	462 (64.1)	101 (64.7)	361 (63.9)	
**Academic Rank**				0.038
Instructor/lecturer/other	92 (12.7)	28 (17.9)	64 (11.3)	
Assistant professor	480 (66.4)	103 (66.0)	377 (66.5)	
Associate or full professor	151 (20.9)	25 (16.0)	126 (22.2)	
**Salary**				0.424
Median (104 k) or below	460 (63.6)	95 (60.9)	365 (64.4)	
Above median	263 (36.4)	61 (39.1)	202 (35.6)	
**Has a doctorate**				0.038
No	625 (86.6)	142 (91.6)	483 (85.2)	
Yes	97 (13.4)	13 (8.4)	84 (14.8)	
**Ever published**				0.135
No	401 (57.0)	93 (62.4)	308 (55.6)	
Yes	302 (43.0)	56 (37.6)	246 (44.4)	
**Received research funding in last 3 years**				0.260
No	631 (87.3)	132 (84.6)	499 (88.0)	
Yes	92 (12.7)	24 (15.4)	68 (12.0)	

*SD* standard deviation

### Satisfaction and burnout

**Fig. 1:**
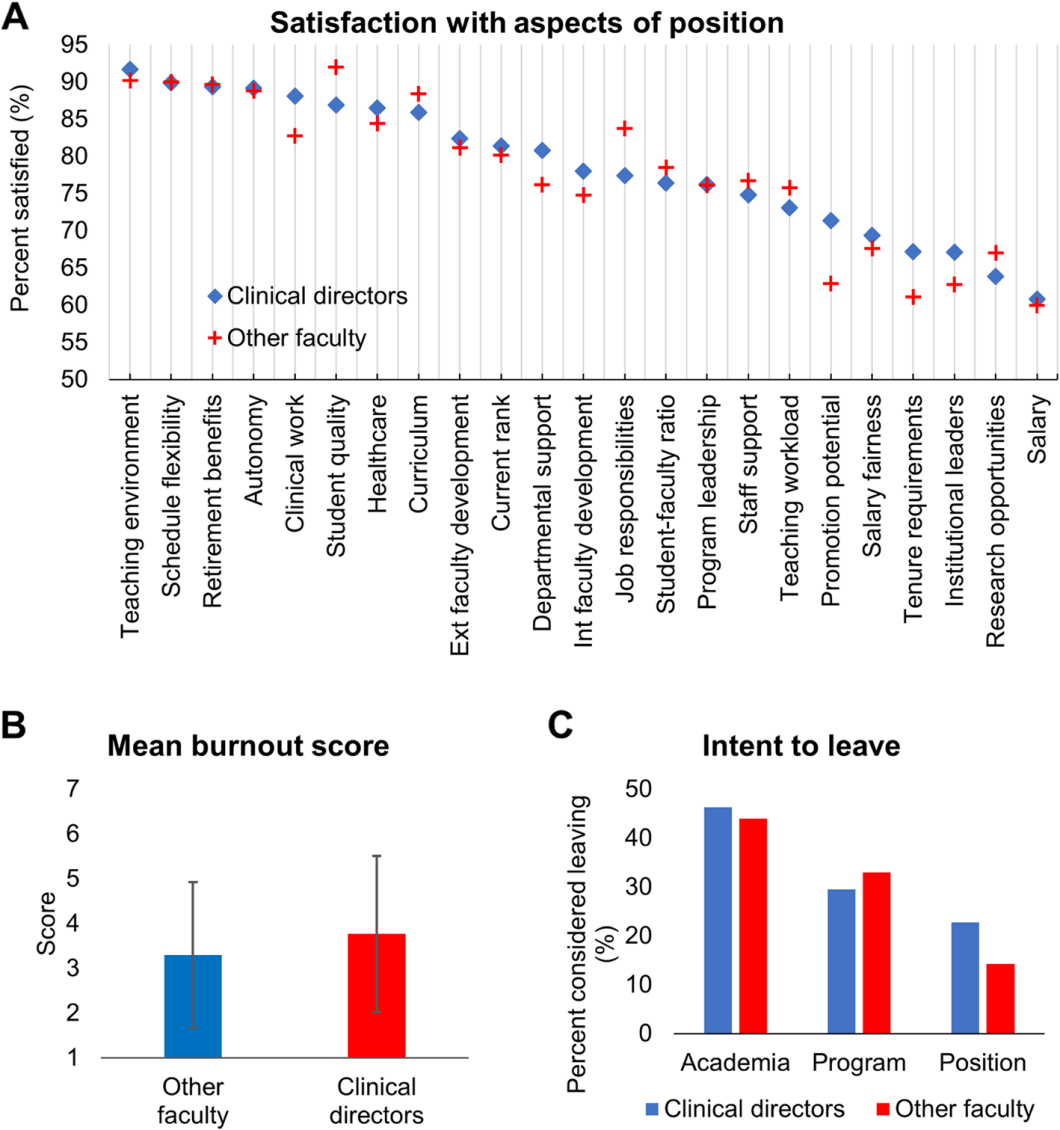
Satisfaction, burnout, and intention to leave among CDs and Other faculty. **A** Percent of CDs and other faculty satisfied with different aspects of their position. **B** Percent of CDs and other faculty who report low, moderate, or high levels of burnout. **C** Percent of CDs and other faculty who report considering leaving academia, their program, or their position

Figure 1A and B summarizes satisfaction and burnout, respectively, for all participants. There were no differences between CDs and other faculty in their satisfaction with any aspect of their position. However CDs had a significantly higher mean burnout score of 3.8 [standard deviation (SD) = 1.7] compared to a mean score of 3.3 (SD = 1.6) for other faculty (*p* = 0.002). Nearly half of all faculty indicated an intent to leave academia (Fig. 1C).

### Faculty considering leaving their position

CDs were almost twice as likely to intend to leave their position as other faculty (Fig. 1C). Table 3 describes predictors of faculty intent to leave their position based on adjusted regression. In CDs, intent to leave was associated with dissatisfaction with professional development, and there was a trend in which each one-point increase in the burnout score increased the odds of leaving by 40% (95% CI, 0.99–1.90). CDs who were dissatisfied with professional development had 3.44 times the odds of intending to leave their position (95% CI, 1.02–11.55). In other faculty, there were no predictors significantly associated with intention to leave their position.

**Table 3:** Multivariable logistic regression for predictors of intention to leave position among CDs and Other faculty

	Considered leaving position							
	Clinical Directors (*n* = 140)				Other Faculty (*n* = 488)			
	AOR^a^	Lower 95% CI	Upper 95% CI	*p* value	AOR^a^	Lower 95% CI	Upper 95% CI	*p* value
***Satisfaction with…***								
**Professional development**								
Satisfied or very satisfied	Ref	Ref	Ref	Ref	Ref	Ref	Ref	Ref
Not satisfied or marginally satisfied	3.44	1.02	11.55	**0.046**	0.94	0.49	1.81	0.850
**Job duties**								
Satisfied or very satisfied	Ref	Ref	Ref	Ref	Ref	Ref	Ref	Ref
Not satisfied or marginally satisfied	0.60	0.12	2.94	0.527	1.33	0.62	2.88	0.464
**Wellbeing & benefits**								
Satisfied or very satisfied	Ref	Ref	Ref	Ref	Ref	Ref	Ref	Ref
Not satisfied or marginally satisfied	0.56	0.10	3.31	0.526	1.41	0.65	3.08	0.386
**Support & environment**								
Satisfied or very satisfied	Ref	Ref	Ref	Ref	Ref	Ref	Ref	Ref
Not satisfied or marginally satisfied	1.67	0.40	6.91	0.479	1.41	0.67	2.97	0.364
**Burnout**	1.37	0.99	1.90	0.056	1.05	0.87	1.25	0.610

*Ref* Reference

^a^Adjusted for age, gender, representation in medicine, years in current position, academic rank, type of school

### Faculty considering leaving their program

Fewer CDs intended to leave their program compared to other faculty (Fig. 1C). Table 4 describes predictors of faculty intent to leave their program. In CDs, every 1-point increase in burnout nearly doubled the odds of considering leaving their program (95% CI, 1.34–2.71). For other faculty, intent to leave was associated with increased levels of burnout (*p* < 0.001), as well as dissatisfaction with professional development (*p* < 0.001) and support and environment (*p* = 0.002).

**Table 4:** Multivariable logistic regression for predictors of intention to leave program among CDs and Other faculty

	Considered leaving program							
	Clinical Directors (*n* = 140)				Other Faculty (*n* = 488)			
	AOR^a^	Lower 95% CI	Upper 95% CI	*p* value	AOR^a^	Lower 95% CI	Upper 95% CI	*p* value
***Satisfaction with…***								
**Professional development**								
Satisfied or very satisfied	Ref	Ref	Ref	Ref	Ref	Ref	Ref	Ref
Not satisfied or marginally satisfied	1.53	0.42	5.57	0.519	3.18	1.91	5.30	**<.001**
**Job duties**								
Satisfied or very satisfied	Ref	Ref	Ref	Ref	Ref	Ref	Ref	Ref
Not satisfied or marginally satisfied	2.25	0.52	9.76	0.280	1.45	0.77	2.74	0.247
**Wellbeing & benefits**								
Satisfied or very satisfied	Ref	Ref	Ref	Ref	Ref	Ref	Ref	Ref
Not satisfied or marginally satisfied	4.68	0.73	29.93	0.103	1.81	0.91	3.60	0.092
**Support & environment**								
Satisfied or very satisfied	Ref	Ref	Ref	Ref	Ref	Ref	Ref	Ref
Not satisfied or marginally satisfied	1.09	0.26	4.49	0.909	2.68	1.45	4.96	**0.002**
**Burnout**	1.90	1.34	2.71	**<.001**	1.39	1.19	1.62	**<.001**

*Ref* Reference

^a^Adjusted for age, gender, representation in medicine, years in current position, academic rank, type of school

### Faculty considering leaving academia

More CDs reported an intent to leave academia compared to other faculty (Fig. 1C). Table 5 describes predictors of faculty intent to leave academia. Each one-point increase in the burnout score increased the odds of CDs considering leaving by almost 3 times (95% CI, 1.80–4.05). A strong association between burnout and intent to leave was also observed in other faculty (AOR = 1.97, 95% CI, 1.67–2.33). Intent to leave academia was associated with increased levels of burnout for both CDs and other faculty. Dissatisfaction with professional development was also significantly associated with intent to leave for other faculty, and showed a non-significant trend with intent to leave for CDs. Dissatisfaction with professional development opportunities increased the odds of other faculty considering leaving academia by 2x (95% CI, 1.24–3.67), while CDs who were dissatisfied with opportunities had nearly 4x the odds of considering leaving academia (95% CI, 0.96–14.95). Dissatisfaction with support and environment was also associated with other faculty’s intent to leave (*p* = 0.0104).

**Table 5:** Results of multivariable logistic regression for predictors of intention to leave academia among CDs and Other faculty

	Considered leaving academia							
	Clinical Directors (*n* = 140)				Other Faculty (*n* = 488)			
	AOR^a^	Lower 95% CI	Upper 95% CI	*p* value	AOR^a^	Lower 95% CI	Upper 95% CI	*p* value
***Satisfaction with…***								
**Professional development**								
Satisfied or very satisfied	Ref	Ref	Ref	Ref	Ref	Ref	Ref	Ref
Not satisfied or marginally satisfied	3.79	0.96	14.95	0.057	2.13	1.24	3.67	**0.006**
**Job duties**								
Satisfied or very satisfied	Ref	Ref	Ref	Ref	Ref	Ref	Ref	Ref
Not satisfied or marginally satisfied	0.75	0.15	3.69	0.728	1.68	0.84	3.33	0.140
**Wellbeing & benefits**								
Satisfied or very satisfied	Ref	Ref	Ref	Ref	Ref	Ref	Ref	Ref
Not satisfied or marginally satisfied	5.78	0.33	101.34	0.230	1.05	0.50	2.20	0.902
**Support & environment**								
Satisfied or very satisfied	Ref	Ref	Ref	Ref	Ref	Ref	Ref	Ref
Not satisfied or marginally satisfied	1.39	0.28	6.76	0.68	2.35	1.19	4.65	**0.014**
**Burnout**	2.70	1.80	4.05	**<.001**	1.97	1.67	2.33	**<.001**

*Ref* Reference

^a^Adjusted for age, gender, representation in medicine, years in current position, academic rank, type of school

## Discussion

Faculty turnover is one current limitation to expanding the PA workforce globally. This study aimed at characterizing CDs and investigating factors that are associated with PA faculty intent to leave in the U.S. The main findings were that nearly half of all PA faculty in the US indicated an intent to leave academia. Among these educators, CDs hold a very important role in coordinating the clinical year of the PA program. Compared to other faculty, CDs generally have worked in PA education, their program, and their position about the same length of time. CDs more often hold the rank of instructor/lecturer and have less doctorate degrees compared to other faculty.

CDs indicated an intention to leave their position almost 2x that of other faculty. Key factors associated with intent to leave were a lack of satisfaction with professional development and burnout. Our results underscore the importance of supporting CDs in reducing burnout and creating more opportunities for professional development and faculty mentoring.

Based on our results, burnout was a factor for CDs intent to leave their program and academia, and likely also their intent to leave their position. Similar observations have been noted in prior studies in the intent to leave and burnout, as well as a relationship between depression, burnout, and professional outcomes among clinically practicing PAs [11–17]. In our analyses, very few other faculty indicated an intention to leave their position, despite 23% reporting feeling burn out at least once a week. Much higher levels reported an intention to leave their program or leave academia altogether.

Another factors strongly associated with intent to leave in both CDs and other faculty was dissatisfaction with professional development opportunities. In CDs, this dissatisfaction contributed to an intention to leave their position and potentially academia, while in other faculty, this dissatisfaction contributed to an intention to leave their program or leave academia. This result may suggest that without a path to advancement, all faculty may intend to leave academia for a different career.

Furthermore, at least 60% of those in each faculty role reported being satisfied with the various aspects of their jobs, suggesting that PA faculty are overall satisfied with their role, despite frequent feelings of burn out. The current study was based on findings pre-pandemic and we have witnessed a major shift in PA burnout rates and resignations. Unique to health profession education, there have been significant challenges in accessing training and clinical sites and preceptors during the COVID-19 pandemic. This has made the job duties of CDs more difficult, so further studies are needed to explore burnout and faculty retention post COVID-19 pandemic.

This study has several implications for academic leaders, employers, faculty and professional associations. Due to the high intent to leave in both CDs and other faculty, there is an urgent need for faculty development, onboarding policies and support for both of these groups. Academic leaders should address the unique challenges associated with the CD role. Potential solutions include reimagining preceptor recruitments to ease the bottlenecks of placing students in clinical training sites.

## Strengths and limitations

The CD is a key member of the PA educational team, and anecdotal reports have indicated high turnover in this group. To our knowledge, this is the first study that focuses on CDs, and thus has the potential to inform efforts for retention. The dataset used surveys all PA faculty in the U.S., ensuring that results are nationally representative. This was a cross-sectional study prior to the pandemic, and PA education, specifically clinical coordination efforts and duties, have changed rapidly during the pandemic, which would likely increase burnout and intent to leave. Because of the cross-sectional nature, it is unknown if the factors associated with intent to leave truly preceded the intent to leave. It is possible that having an intent to leave may contribute to an individual’s perception of their workplace or impact their interactions in a way that could influence factors such as satisfaction. Additionally, because PA programs typically have one CD but many faculty, the number of CDs in the study was on the smaller size, which reduces the power of our analyses. The study was sufficiently powered to identify several significant associations; however, with a larger sample, some of the additional trends we observed may have reached significance. The main outcome variable measured intent to leave, but how this intention translates into actual faculty turnover remains to be investigated through longitudinal studies. This study focused on PA faculty from the U.S. but we believe the findings are generalizable to other regions. The study was based on survey research and secondary data analysis which have inherent self-selection bias, and self reported responses.

## Conclusions

Faculty turnover is a major limiting factor in PA education as approximately 50% of all PA faculty intend to leave academia in the next 2 years. We found that clinical coordinators in particular are more likely to intend to leave compared to other faculty. In both CDs and other faculty, intent to leave was significantly associated with a lack of satisfaction with professional development, and moderate to high levels of burnout; however, only in CDs were these specifically associated with intention to leave their current position. This study underscores the importance of mentoring and providing faculty development opportunities to the entire PA professoriate. Longitudinal and qualitative research are needed to further investigate the unique characteristics of CDs.

## Data Availability

The datasets used and/or analyzed during the current study are available from the corresponding author on reasonable request.

## Abbreviations

AAPA: American Academy of Physician Assistants; Physician Assistant/Associate
PAEA: Physician Assistant Education Association
FTE: Fulltime equivalent
CD: Clinical Directors
PA: Physian Assistant/Associate

## Footnotes

1 May also be referred to as Clinical Coordinators

## References

[1] Gordes KL, Fleming SC, Cawley JF, Kulo V (2021). Advancing physician assistant faculty development: a new model. J Physician Assist Educ.

[2] Physician assistant education association, by the numbers: program report 35: data from the 2019 program survey. Washington, DC: PAEA; 2020. 10.17538/FR4.2020.

[3] Min EA, Bowden WP, Gerstner LR, Guthrie JR (2021). Credentials and Core Clerkships: Who's Training Our Physician Assistant Students (and Does It Matter)?. J Physician Assist Educ.

[4] Hills K, VanderMeulen S, Snyder JA, Kohlhepp W, Alexander LM, Lane S (2020). Reimagining physician assistant education. J Physician Assist Educ.

[5] Physician Assistant Education Association (2018). By the numbers: curriculum report 3: data from the 2017 clinical curriculum survey.

[6] Physician Assistant Education Association (2013). Payment of clinical sites and preceptors in PA education. Issue Brief. From Clerkship Survey.

[7] Brown D, Sivahop JN (2017). Challenges of Clinical Education. J Physician Assist Educ.

[8] Snyder JA, Lucich JA, Zorn JS, Enking PJ, Barnett JS, Fahringer D (2010). Clinical coordination and the experiential year in physician assistant education. J Physician Assist Educ.

[9] Kim S, Park SM (2014). Determinants of job satisfaction and turnover intentions of public employees: Evidence from US federal agencies. Int Rev Public Admin.

[10] Holland JI. Making vocational choices: A theory of careers. Englewood Cliffs: Prentice Hall; 1973.

[11] Blackstone SR, Johnson AK, Smith NE, McCall TC, Simmons WR, Skelly AW (2021). Depression, burnout, and professional outcomes among PAs. JAAPA..

[12] Geurts S, Schaufeli W, De Jonge J (1998). Burnout and intention to leave among mental health-care professionals: a social psychological approach. J Soc Clin Psychol.

[13] Cantu R, Carter L, Elkins J. Burnout and intent-to-leave in physical therapists: a preliminary analysis of factors under organizational control. Physiother Theory Pract. 2022;38(13):2988-97. 10.1080/09593985.2021.1967540 34429016

[14] Beltyukova S, Graham K (2017). Predictors of physician assistant faculty intent to leave academia: a Rasch regression analysis. J Physician Assist Educ.

[15] Graham K, Beltyukova S (2015). Development and initial validation of a measure of intention to stay in academia for physician assistant faculty. J Physician Assist Educ.

[16] Coniglio D, Akroyd D (2015). Factors predicting physician assistant faculty intent to leave. J Physician Assist Educ.

[17] Hooker RS, Kuilman L, Everett CM (2015). Physician assistant job satisfaction: a narrative review of empirical research. J Physician Assist Educ.

